# Time trend in the surgical management of obstructed defecation syndrome: a multicenter experience on behalf of the Italian Society of Colorectal Surgery (SICCR)

**DOI:** 10.1007/s10151-022-02705-x

**Published:** 2022-09-14

**Authors:** A. Picciariello, M. Rinaldi, U. Grossi, M. Trompetto, G. Graziano, D. F. Altomare, SICCR ODS Study Group, G. Gallo

**Affiliations:** 1 Department of Emergency and Organ Transplantation and Inter-Department Research Center for Pelvic Floor Disease (CIRPAP), University Aldo Moro of Bari, Bari, Italy; 2 Department of Surgery, Oncology and Gastroenterology–DISCOG, University of Padua, Padua, Italy; 3 Department of Colorectal Surgery, S. Rita Clinic, Vercelli, Italy; 4 Center for Outcomes Research and Clinical Epidemiology (CORESEARCH), Pescara, Italy; 5 Department of Surgical Sciences, La Sapienza” University of Rome, Rome, Italy

**Keywords:** Obstructed defecation syndrome, ODS, Constipation, Transanal, Rectopexy

## Abstract

**Background:**

Surgical management of obstructed defecation syndrome (ODS) is challenging, with several surgical options showing inconsistent functional results over time. The aim of this study was to evaluate the trend in surgical management of ODS in a 10-year timeframe across Italian referral centers.

**Methods:**

Surgeons from referral centers for the management of pelvic floor disorders and affiliated to the Italian Society of Colorectal Surgery provided data on the yearly volume of procedures for ODS from 2010 to 2019. Six common clinical scenarios of ODS were captured, including details on patient’s anal sphincter function and presence of rectocele and/or rectal intussusception. Perineal repair, ventral rectopexy (VRP), transanal repair (internal Delorme), stapled transanal rectal resection (STARR), Contour Transtar, and transvaginal repair were considered in each clinical scenario.

**Results:**

Twenty-five centers were included providing data on 2943 surgical patients. Procedure volumes ranged from 10–20 (54%) to 21–50 (46%) per year across centers. The most performed techniques in patients with good sphincter function were transanal repair for isolated rectocele (243/716 [34%]), transanal repair for isolated rectal intussusception (287/677 [42%]) and VRP for combined abnormalities (464/976 [48%]). When considering poor sphincter function, these were perineal repair (112/194 [57.8%]) for isolated rectocele, and VRP for the other two scenarios (60/120 [50%] and 97/260 [37%], respectively). The use of STARR and Contour Transtar decreased over time in patients with impaired sphincter function.

**Conclusions:**

The complexity of ODS treatment is confirmed by the variety of clinical scenarios that can occur and by the changing trend of surgical management over the last 10 years.

## Introduction

More than 30% of patients suffering from chronic constipation have obstructed defecation syndrome (ODS). Obstructive phenomena can result from structural and/or functional causes that eventually impede stool expulsion [1, 2].

Hard stool, straining, incomplete evacuation, bloating, and abdominal discomfort are the most frequently reported complaints, affecting patients’ quality of life and causing a significant psychological burden [3].

Symptom relief can be achieved with first-line therapies, including behavioral and conservative interventions, manipulating the microbiome, and pharmacological treatments [4, 5].

However, surgery is still the mainstay of treatment for almost 25% of patients with ODS who fail conservative approaches [4, 6].

The surgical management of ODS has changed in the last two decades, with several operative techniques encompassing a large spectrum of anatomical approaches [7–13].

Nevertheless, a mixture of functional and anatomical abnormalities is often responsible for complexity of this disorder [14], with several contributing factors that cannot be addressed by surgery alone. Therefore it is unrealistic to hope to develop a procedure that works for all patients.

This study evaluated the trend of different surgical approaches for the treatment of ODS patients recruited from Italian referral centers in a 10-year timeframe.

## Materials and methods

Surgeons affiliated to the Italian Society of Colorectal Surgery (SICCR) were selected based on their expertise in the surgical management of ODS (from centers performing at least 10 procedures per year), favoring those with track record in research and publications in the field of pelvic floor disorders. Surgeons were invited to fill out an Excel spreadsheet including the number and type of procedures for ODS performed each year during the period between 1st January 2010 and 31st December 2019. Participants were also asked to complete a preliminary questionnaire exploring the ODS diagnostic work-up.

Six common clinical scenarios of ODS were proposed, including details on anal sphincter function and the presence of specific anatomical abnormalities (e.g. rectocele and/or rectal intussusception) in isolation or combined.

For each clinical scenario, the six most frequently performed surgical techniques were considered: perineal repair, ventral rectopexy (VRP; via open or laparoscopic/robotic approach), transanal repair (internal Delorme [15]), stapled transanal rectal resection (STARR), Contour Transtar, and transvaginal repair. Combined approaches were not considered for the analysis.

### Statistical analysis

Data were reviewed and summarized in terms of percentages. Trends over the study period were analyzed. Comparisons between multiple groups of procedures and their association with time were performed using univariate multinomial logistic regression models, separately for each ODS subtype. The results were presented as odds ratios (ORs) with 95% confidence intervals (CIs). The predicted probabilities calculated from the models were also reported in specific graphs. All analyses were performed using the Statistical Analysis System (SAS) Package, Release 9.4 (SAS Institute, Cary, North Carolina, USA).

## Results

Twenty-five principal investigators affiliated to the SICCR from 25 centers were included in this study providing data on 2943 operated patients. All centers completely filled out each part of the Excel database. After the preliminary questionnaire, 94% of the investigators agreed that X-ray and/or magnetic resonance imaging defecography are mandatory to establish a surgical indication in ODS patients. Similarly, the use of preoperative scoring systems to determine the severity of ODS was supported (‘considered useful’) by 70% of the participants for surgical decision-making. Procedure volumes ranged from 10–20 (54%) to 21–50 (46%) per year across centers.

## Good sphincter function

### Scenario 1 – Isolated rectocele in patients with good sphincter function

**Fig. 1:**
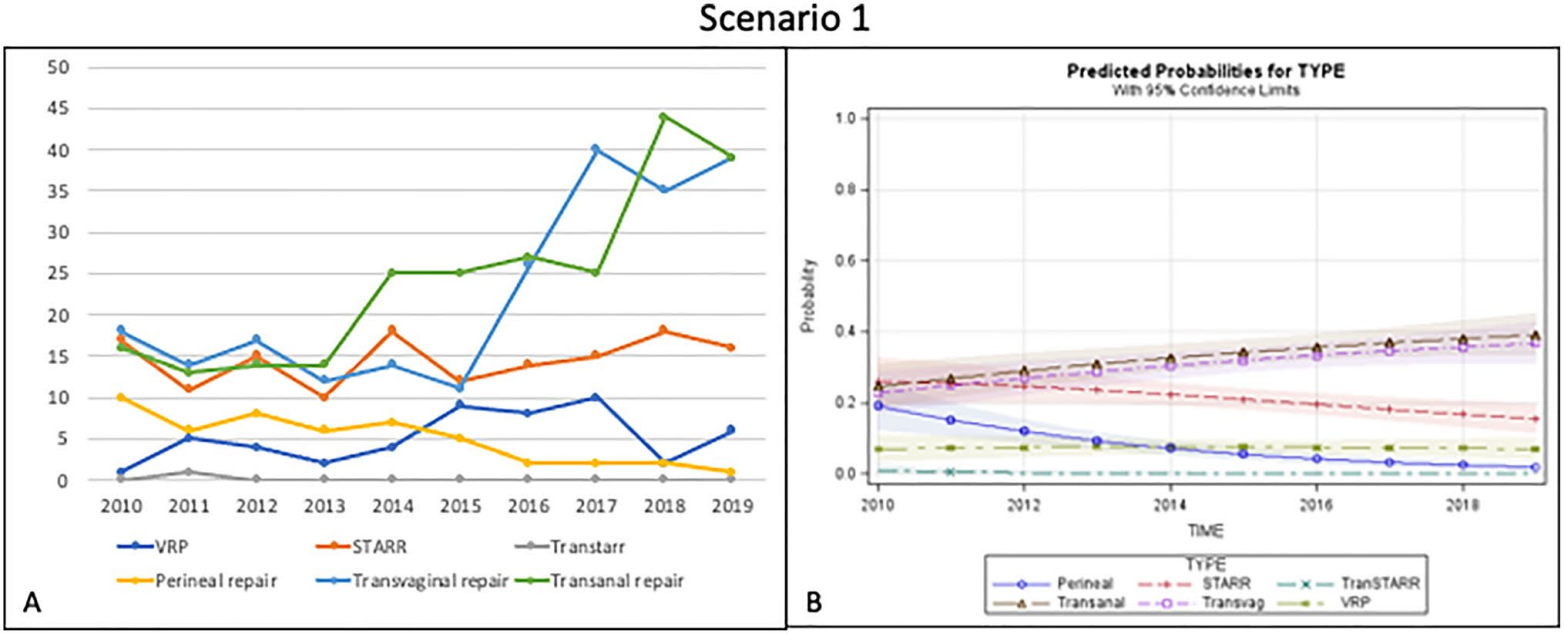
Trend of the different surgical procedures over time (**a**) and predicted probability model (**b**) for Scenario 1 (good sphincter function with rectocele)

Among the 716 patients included in this scenario, transanal repair (34%) was the most performed technique, showing an increasing trend over time, especially during the last 3 years. Transvaginal repair was performed in 31.5% of cases, and STARR in 20.3%, showing a constant trend over the last 5 years. Perineal repair and VRP were performed in 6.9% and 7.2% of patients, respectively, while Contour Transtar was adopted in only 0.1% of cases (Fig. 1a).

When the frequency of each operation was plotted against time, the predicted probability analysis showed a linear increase of transanal and transvaginal approaches, the use of STARR and perineal repair decreased progressively, and VRP remained constantly limited (Fig. 1b).

### Scenario 2 – Isolated rectal intussusception in patients with good sphincter function

**Fig. 2:**
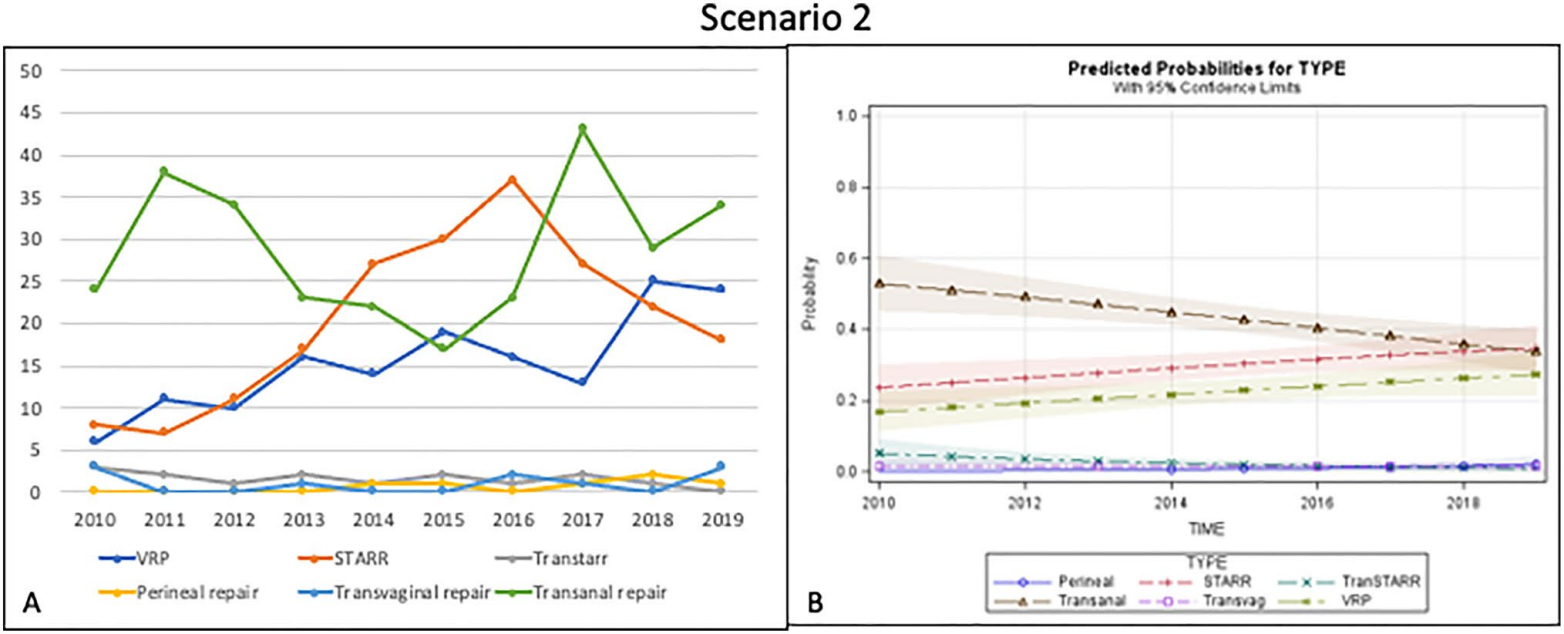
Trend of the different surgical procedures over time (**a**) and predicted probability model (**b**) for Scenario 2 (good sphincter function with rectal intussusception)

In this scenario, 677 patients were recruited. Almost half (42.4%) were treated by transanal repair. STARR was performed in 30.1% of cases, followed by VRP (22.8%). Smaller percentages of patients were treated by Contour Transtar (2.3%), transvaginal repair (1.5%) and perineal repair (0.9%) (Fig. 2a).

For this scenario, the predicted probability analysis showed a significant reduction of the transanal approach and a slow but progressive and parallel increase in STARR and VRP. Perineal and transvaginal approaches seemed to have no indication in this scenario while Contour Transtar was rarely performed (Fig. 2b).

### Scenario 3 – Rectocele and rectal intussusception in patients with good sphincter function

**Fig. 3:**
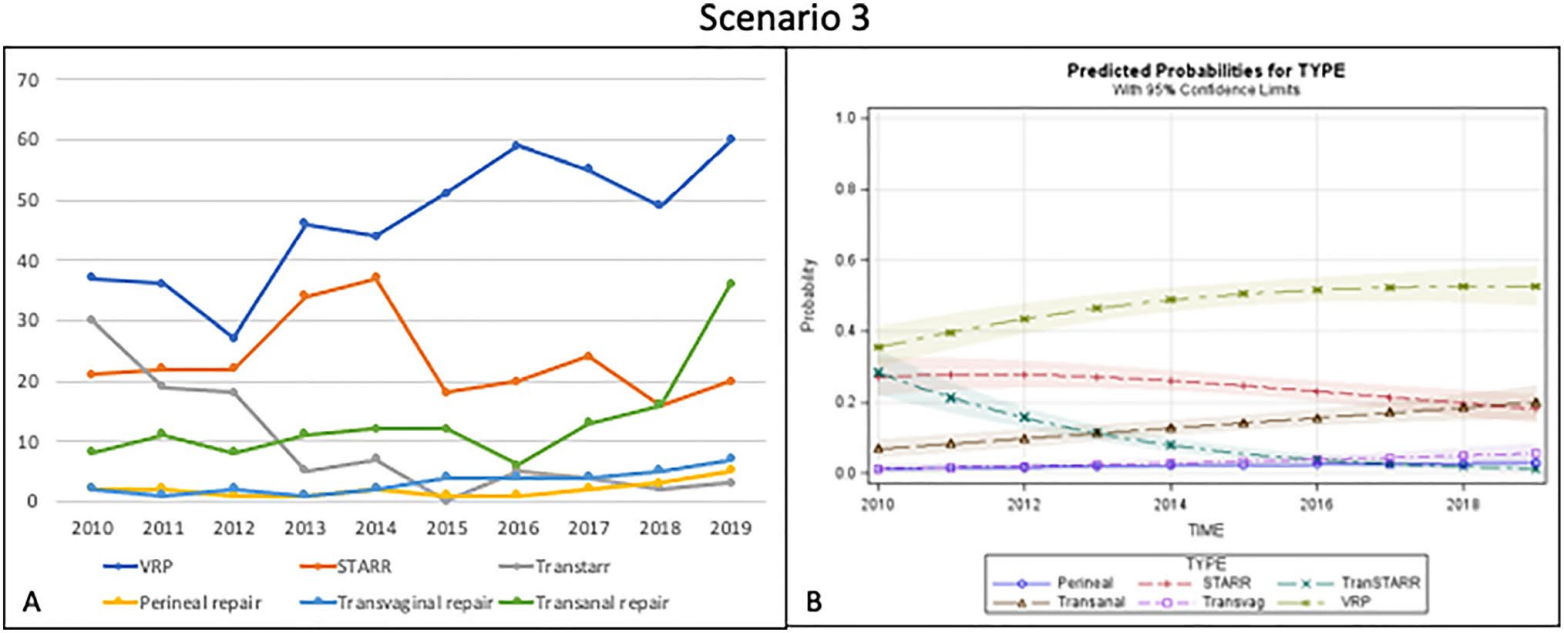
Trend of the different surgical procedures over time (**a**) and predicted probability model (**b**) for Scenario 3 (good sphincter function with rectocele and L rectal intussusception)

Considering the 976 patients in this scenario, the most frequently performed technique was the VRP (47.5%), increasing by more than 50% over the last 5 years. STARR was carried out in 24% of patients and transanal repair in 13.7%. Contour Transtar showed a decreasing trend during the last 3 years (falling to 9.5% of cases), while transvaginal and perineal repair were performed in 3.3% and 2% of patients, respectively (Fig. 3a).

The predicted probability analysis in this scenario indicated a clear and constant increase for VRP and moderate increase of the transanal approach, paralleled by a decline in STARR and Contour Transtar techniques (Fig. 3b).

## Poor sphincter function

### Scenario 4 – Isolated rectocele in patients with poor sphincter function

**Fig. 4:**
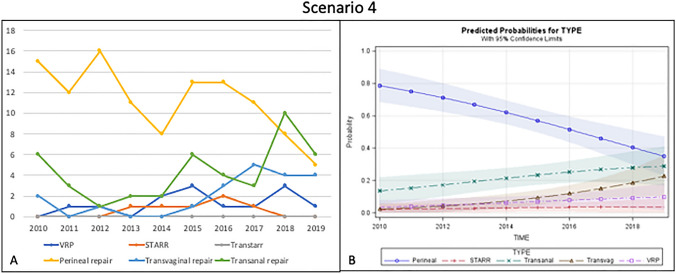
Trend of the different surgical procedures over time (**a**) and predicted probability model (**b**) for Scenario 4 (poor sphincter function with rectocele)

Overall, 194 patients were included in this scenario. The most frequently adopted treatment was the perineal repair (57.8%), followed by transanal (22.1%) and transvaginal (10.4%) repairs. VRP was performed only in 13 patients (6.7%)and STARR in 6 cases (3%) (Fig. 4a).

However, when considering the time trend in the predicted probability analysis, the choice of perineal approach showed a steep decrease, while transanal and transvaginal approaches slowly increased. VRP and STARR were rarely performed in this scenario (Fig. 4b).

### Scenario 5—Isolated rectal intussusception in patients with poor sphincter function

**Fig. 5:**
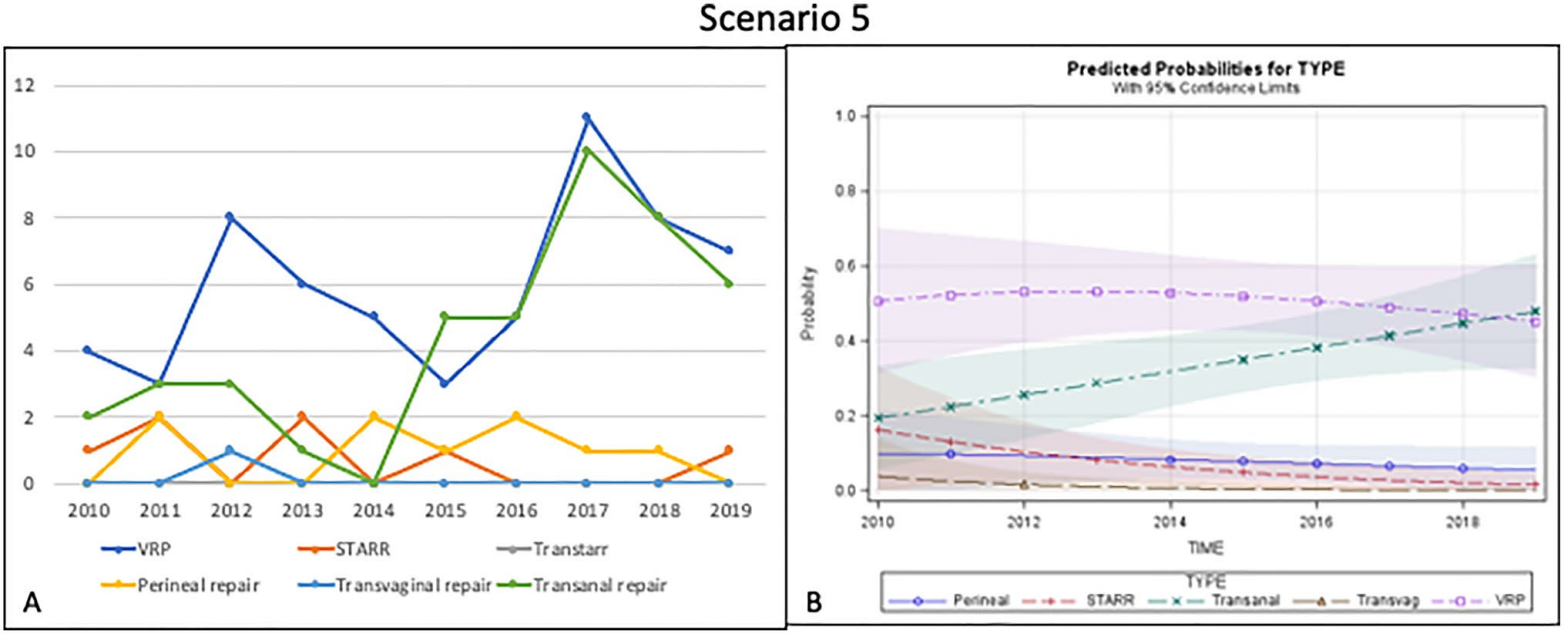
Trend of the different surgical procedures over time (**a**) and predicted probability model (**b**) for Scenario 5 (poor sphincter function with rectal intussusception)

In total, 120 patients were included. Half of them underwent VRP, while transanal and perineal repairs were performed in 36% and 7.5% of cases, respectively. Transvaginal repair was carried out in only 1 (0.8%) patient and STARR in 7.5% of cases (Fig. 5a).

The predicted probability model showed that VRP is still the most frequently adopted technique even if transanal repair has increasingly been performed in recent years, while STARR was used infrequently, and the other options very rarely considered (Fig. 5b).

### Scenario 6 – Rectocele and rectal intussusception in patients with poor sphincter function

Out of 260 patients included in this scenario, 97 (37.4%) underwent VRP, showing an increasing trend over the last 5 years. The use of STARR declined in the last 3 years and was performed overall in 13% of cases. Only 4.3% of patients underwent Contour Transtar.

**Fig. 6:**
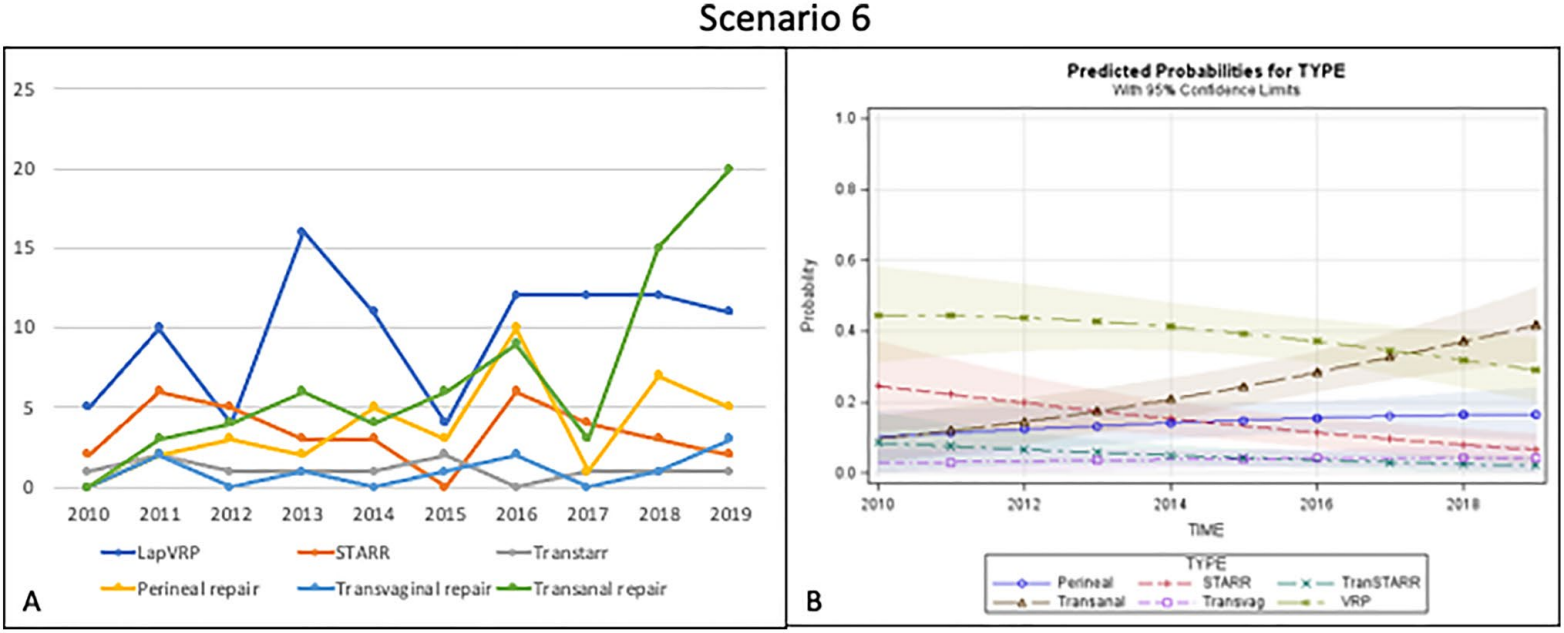
Trend of the different surgical procedures over time (**a**) and predicted probability model (**b**) for Scenario 6 (poor sphincter function with rectocele and rectal intussusception)

Transanal, perineal and transvaginal repairs were carried out in 26.9%, 14.6%, and 3.8%, respectively (Fig. 6a).

The predicted probability analysis showed that VRP was the preferred operation in this scenario, but there was an increasing trend in transanal repair. The choice of STARR decreased progressively. The perineal approach was still an option in a minority of cases, and its frequency was maintained over time. Finally, there was no indication for transvaginal and Contour Transtar operations in this scenario (Fig. 6b).

## Discussion

In the last two decades, several surgical options have been proposed for the management of ODS patients showing inconsistent functional results over time. Although some studies have compared the outcomes of different techniques [16, 17], there is still a paucity of data on the evolution and time trend of surgical choices in the treatment of ODS. Although limited to Italy, our study partially overcomes this knowledge gap by providing data on a large cohort from 25 referral centers over a 10-year period.

Almost all participants supported the use of defecography as first-line diagnostic modality in patients with refractory constipation, in line with previous recommendations [14]. Rectocele and/or rectal intussusception were selected for clinical scenarios because these are the most common anatomical abnormalities in patients with moderate-to-severe symptoms of constipation [2].

The use of a dedicated scoring system, which is mandatory in other countries for the accreditation of a Pelvic Floor Unit, is limited to 70% of the centers involved in the study suggesting that more work is needed to increase its use in the evaluation of the patients’ condition.

The 10-year-old AIGO/SICCR consensus statement highlighted controversial results of two surgical approaches (i.e. abdominal [rectopexy] and transanal [STARR or Delorme transrectal excision]) to correct rectal intussusception, while supporting transanal, transvaginal, and perineal routes for rectocele repair [5].

Nevertheless, at the time of this consensus, sphincter function and the possible association of multiple anatomical abnormalities were not taken into account for the decision-making process.

According to the recently published European E-consensus guidelines [18], the choice of treatment is strictly related to the clinical scenario, with sphincter function as one of the key drivers. Considering that several scenarios can be observed in current practice, encompassing a wide spectrum of abnormalities, surgeons dedicated to the treatment of pelvic floor disorders are expected to be able to perform operations using abdominal, transanal, transvaginal and/or perineal approaches, whenever indicated.

In this study, most patients with rectocele and good sphincter function underwent transanal, followed by transvaginal approaches, while the perineal approach was performed in a limited group of patients. The use of the STARR technique, reported in about 20% of the cases, is still the preferred option in a few colorectal units well trained in this procedure. This trend only partially reflects the algorithm recently proposed by the European Consensus on ODS [18], indicating the transanal approach as third choice treatment after transvaginal and perineal repair. Unlike this statement, our survey shows that most Italian colorectal surgeons still prefer the transanal approach to repair an isolated rectocele.

A recent systematic review [19] suggests that perineal rectocele repair could be an effective method for symptom relief with a complication rate similar to that of transanal rectocele repair. The latter approach was also shown to be effective in improving constipation-specific quality of life in patients with rectocele [20].

In case of rectocele combined with rectal intussusception irrespective of anal sphincter function, our data showed that laparoscopic VRP was the most frequently performed technique, especially over the last 5 years. Conversely, popularity of STARR and Contour Transtar significantly declined over the last 3 years.

VRP has gained favor amongst colorectal surgeons as an operation able to improve bowel symptoms by simultaneous correction of multiple anatomical abnormalities. A previous consensus statement highlighted the advantage of the laparoscopic ventral approach in the improvement of constipation compared to posterior rectopexy [21]. VRP can be performed by minimally invasive techniques, including both robotic and laparoscopic approaches. In fact, according to our study, the laparoscopic approach has become more frequent over time, with the open approach limited to very selected cases over the last few years.

In the first prospective multicenter trial on STARR for ODS, Boccasanta et al. [22] reported good short-term results in approximately 90% of the patients, but painful defecation occurred at 1 year in 20%. Several subsequent studies have reported various complications after STARR including proctalgia, bleeding, urgency, incontinence, pain, constipation, and rectovaginal fistula [23–26].

A recent retrospective study demonstrated that failure of the STARR procedure for ODS could be due to persistence or de novo alteration of the anorectal anatomy on defecation. Nevertheless, in 40% of patients complaining of incomplete emptying or incontinence after STARR, no anatomical abnormalities were observed [27].

When rectocele is associated with impaired sphincter function, our study indicated that transanal and transvaginal routes were the preferred approaches over time, with a decrease of the perineal approach and a slight increase in transabdominal operations. This trend is at odds with the European consensus [18] and does seem illogical since a transanal approach could potentially further damage the anal sphincter. In fact, in the European consensus concerning the treatment of patients with ODS and impaired anal sphincter a full agreement (100%) for the use of VRP was achieved.

STARR was rarely performed in all scenarios with impaired sphincter function, while VRP and transanal repair represented the most frequently performed techniques in case of rectal intussusception in isolation or combined with rectocele. Laparoscopic VRP has been shown to be safe and effective in this scenario, with an acceptable morbidity rate [28–30]. When compared to STARR, VRP give a superior outcome if overall pelvic floor function is considered [16]. A recent randomized clinical trial on elderly patients showed that even in the presence of comorbidities, laparoscopic VRP yields better long-term functional outcomes, less complications and recurrences compared to STARR [31]. A French study showed that laparoscopic VRP represents a valid alternative to STARR in patients with anal sphincter weakness for the treatment of outlet obstruction associated with recto-anal intussusception and rectocele[32].

Limitations of this study include its retrospective nature and the involvement of only Italian centres and centers carrying out only 10–20 procedures per year.

## Conclusions

The complexity of ODS treatment is confirmed by the variety of clinical scenarios that can occur and by the changing trend of surgical management over the last 10 years. The choice of procedure should be driven by the clinical scenario and sphincter function. The Italian experience partially reflects the recently published European guidelines, even if some controversial aspects still need to be clarified.
